# Aichivirus a Infection Associated with Severe Enteric and Respiratory Disease in a Captive White Lion (*Panthera leo*)

**DOI:** 10.3390/microorganisms14102256

**Published:** 2026-10-04

**Authors:** Yushan Ding, Yajiang Wu, Fen Shan, Niu Zhou, Mengna Lv, Chen Wang, Minting Liu, Wanping Li, Wu Chen, Zujin Chen

**Affiliations:** 1 Guangzhou Zoo & Guangzhou Wildlife Research Center, Guangzhou 510070, China; dingyushan0517@163.com (Y.D.); yajiangwu@126.com (Y.W.); shanfen_gzzoo@sina.com (F.S.); zhouniu@zju.edu.cn (N.Z.); m18819266782@163.com (M.L.); conan1996728@hotmail.com (M.L.); tabitha313@outlook.com (W.L.); 2 College of Veterinary Medicine, South China Agricultural University, Guangzhou 510642, China

**Keywords:** kobuvirus, *Panthera leo*, transcriptomic analysis, cross-species transmission

## Abstract

Kobuviruses are emerging enteric pathogens with broad host ranges, yet their pathogenic potential in wild large felids remains largely unexplored. Here, we report the first case of severe enteric and respiratory disease associated with AiV-A infection in a large felid, designated FeKoV0813, in a captive juvenile white lion (*Panthera leo*). The animal presented with acute respiratory distress and severe diarrhea, culminating in death despite supportive care. Comprehensive diagnostic workflows, including necropsy, histopathology, fluorescence in situ hybridization (FISH), and absolute quantitative RT-PCR, were employed to determine tissue tropism and viral dissemination. Whole-genome sequencing, phylogenetic analysis, and selection pressure assessment were conducted to characterize the viral evolutionary dynamics and host-specific constraints. The 8213-nucleotide genome exhibited typical Kobuvirus organization, clustering within the AiV-A clade alongside carnivore-derived strains from dogs, cats, and foxes. High viral RNA loads were detected primarily in the small intestine (~7.4 log_10_ copies/100 ng RNA) and trachea, with dissemination to the kidney and pancreas. Histopathological analysis revealed epithelial lesions, and FISH localized viral RNA to these affected epithelial cells, while transcriptomic profiling revealed higher transcript abundance of *CXCL8* and other pro-inflammatory genes in the intestine than in other tissues. Notably, selection pressure analysis indicated strong purifying selection acting on the feline *VP1* gene, with no positively selected sites identified, contrasting with signatures of episodic positive selection observed in human and rodent lineages. This study provides the first report of severe respiratory and gastrointestinal disease associated with AiV-A infection in large felids, significantly expanding the known host range of kobuviruses. Our findings underscore the need for vigilant surveillance of kobuviruses in captive wildlife and highlight the potential risk of spillover infections from domestic carnivore reservoirs.

## 1. Introduction

The genus *Kobuvirus*, within the family *Picornaviridae*, comprises genetically diverse, non-enveloped viruses with positive-sense, single-stranded RNA genomes. Kobuviruses are primarily associated with enteric infection, although extra-intestinal dissemination has been reported in some hosts [1,2]. According to the International Committee on Taxonomy of Viruses (ICTV), the genus currently comprises six species, designated AiV-A-F [3]. Among these, AiV-A has been identified in a broad range of hosts, including humans, domestic carnivores, livestock, birds, and rodents [4,5,6,7,8,9].

The AiV-A genome is approximately 8.2–8.4 kb in length, consisting of a 5′ untranslated region (UTR), a single polyprotein-encoding open reading frame (ORF) organized as L-VP0-VP3-VP1-2A-2B-2C-3A-3B-3C-3D, and a 3’ UTR [10]. VP1, a surface-exposed capsid protein, contributes to host-cell recognition. Structural studies of human AiV have identified a C-terminal polyproline helix within VP1 that may participate in receptor interactions, suggesting a potential role for capsid architecture in determining host or cellular tropism [11].

In domestic carnivores, AiV-A has been detected predominantly in fecal samples from dogs and cats with enteric disease, with reported prevalence varying among populations [12,13,14,15]. Its contribution to disease, however, remains incompletely defined. Evidence from wild carnivores further indicates that kobuvirus infection may extend beyond the gastrointestinal tract. In a fox co-infected with canine distemper virus, canine kobuvirus RNA was detected in peripheral tissues and the central nervous system [16,17].

White lions carry a recessive mutation in the *TYR* gene [18], and captive breeding to maintain this phenotype involves inbreeding that is associated with compromised immune function, including reduced MHC diversity and impaired cellular and humoral immunity [19]. Additional congenital defects linked to the leucistic allele include cleft palate, spinal deformities, cardiac and renal defects, and lung developmental abnormalities [20]. The potential immunological susceptibility of this animal may have contributed to disease severity.

This study addresses a key gap in knowledge by delineating the histopathological and tissue-distribution features of AiV-A infection in a white lion and by interrogating whether AiV-A alone can cause multi-organ damage and death in large felids without co-infection. Here, we describe FeKoV0813, an AiV-A strain identified in a captive juvenile white lion that presented with acute respiratory distress, severe diarrhea, and anorexia and subsequently died. Post-mortem metatranscriptomic and molecular analyses detected viral RNA in multiple tissues, with the highest loads in the small intestine and trachea. Histopathology, quantitative RT-PCR, fluorescence in situ hybridization, whole-genome sequencing, and host transcriptomic profiling were used to characterize viral distribution, tissue involvement, host responses, and the evolutionary relationship of FeKoV0813 to other AiV-A strains. Because this is a single fatal case and the virus was not isolated, a definitive answer to whether AiV-A alone caused multi-organ damage and death cannot be established; nevertheless, this case extends the known host range of AiV-A to a large felid and provides evidence of multi-tissue viral involvement in a fatal disease case, establishing a basis for further investigation of AiV-A infection in captive and wild felids.

## 2. Materials and Methods

### 2.1. Animal and Sample Collection

A captive juvenile white lion (*Panthera leo*) was housed at a zoological facility operated by a wildlife conservation agency in Henan Province, China. The animal was an 8-month-old male born in captivity at the facility. It had no history of prior illness and had received routine vaccinations against feline panleukopenia, feline herpesvirus, and feline calicivirus approximately three months before disease onset. It was fed a commercially prepared raw meat-based diet supplemented with vitamins and minerals, with no recent dietary changes. It was housed individually, and no contact animals were available for sampling; consequently, no contact-animal swabs could be collected for viral screening. The lion presented with respiratory signs, including wheezing and coughing, together with anorexia and severe diarrhea. Despite supportive treatment, it died 20 days after the onset of clinical signs. A complete necropsy was performed immediately after death.

Samples of the trachea, lung, heart, kidney, liver, intestine, and pancreas were collected. For histopathological examination and in situ hybridization, tissues were fixed in 4% paraformaldehyde for 24–48 h at 4 °C. Parallel tissue samples were snap-frozen in liquid nitrogen and stored at −80 °C until RNA extraction and subsequent molecular analyses. All procedures were conducted in accordance with institutional animal welfare guidelines.

### 2.2. RNA Extraction and Metatranscriptomic Sequencing

Total RNA was extracted from tissue samples using TRIzol Reagent (Invitrogen Life Technologies, Carlsbad, CA, USA). RNA concentration and quality were assessed using a NanoDrop spectrophotometer (Thermo Scientific, Waltham, MA, USA), and 3 μg of total RNA per sample was used for library preparation. Poly(A)+ RNA was enriched using poly-T oligo-attached magnetic beads and fragmented prior to first- and second-strand cDNA synthesis. Double-stranded cDNA was end-repaired, 3′-adenylated, ligated to Illumina paired-end adapters, and size-selected to obtain fragments of approximately 400–500 bp using the AMPure XP system (Beckman Coulter, Brea, CA, USA). Libraries were amplified for 15 PCR cycles, purified, and quantified using the Agilent High Sensitivity DNA Assay on a Bioanalyzer 2100 system (Agilent Technologies, San Diego, CA, USA). The final libraries were sequenced on an Illumina NovaSeq 6000 platform at Shanghai Personal Biotechnology Co., Ltd., Shanghai, China.

### 2.3. Metatranscriptomic Screening for Co-Infecting Pathogens

To investigate the presence of potential co-infecting pathogens, a retrospective metatranscriptomic analysis was performed using the sequencing dataset deposited under BioProject accession number PRJNA1510436. Raw sequencing reads were quality-filtered using fastp v0.23.2 to generate clean reads.

For bacterial profiling, clean reads were analyzed using MetaPhlAn v4.0.6 with its marker-gene database. For broader pathogen screening, clean reads were assembled de novo into contigs using MEGAHIT v1.2.9. The resulting contigs were searched against the NCBI non-redundant (nr) protein database, the Fungi ITS RefSeq database, and the MIDORI2 database, which contains curated reference sequences from diverse eukaryotic taxa, to identify sequences of potential viral, fungal, and parasitic origin [21,22].

### 2.4. Histopathology

Tissue samples from the trachea, lung, kidney, liver, heart, pancreas, and intestine were fixed in 4% paraformaldehyde at 4 °C for 24–48 h, dehydrated through a graded ethanol series, cleared in xylene, and embedded in paraffin. Paraffin-embedded tissues were sectioned at 4 μm, mounted on glass slides, deparaffinized, rehydrated, and stained with hematoxylin and eosin (H&E) according to standard procedures. Histological sections were examined using an Olympus BX53 light microscope (Olympus, Tokyo, Japan).

Histopathological evaluation focused on epithelial integrity, inflammatory cell infiltration, necrosis, and glandular alterations, where applicable. Serial sections were prepared on RNase-free slides for subsequent in situ hybridization.

### 2.5. Dual-Fluorescence In Situ Hybridization (FISH)

Paraffin-embedded tissue sections (4 μm) from the trachea, kidney, small intestine, and rectum were mounted on RNase-free, poly-L-lysine-coated slides. Sections were deparaffinized in xylene, rehydrated through a graded ethanol series, and subjected to heat-mediated antigen retrieval in citrate buffer (pH 6.0) at 95 °C for 15 min. Sections were then digested with proteinase K (20 μg/mL) at 37 °C for 20 min, post-fixed in 4% paraformaldehyde for 10 min, and acetylated with 0.25% acetic anhydride in 0.1 M triethanolamine for 10 min.

Fluorescence in situ hybridization (FISH) was performed using a commercial kit (Servicebio, Wuhan, China) according to the manufacturer’s instructions [23]. A probe targeting a conserved region of the *kobuvirus VP1* gene (5′-CCAGACCAGTCCTCCCACCCGTAG-3′) was synthesized by Servicebio; probe sequences are provided in Table S1. This was validated by a BLAST + (v2.17.0) search against the NCBI database to ensure no predicted cross-reactivity with other viruses. Hybridization was performed overnight at 42 °C in a humidified chamber. Sections were subsequently subjected to stringent washes in 2× saline sodium citrate (SSC) at 42 °C and 0.5× SSC at 60 °C. Nuclei were counterstained with DAPI, and images were acquired using an Olympus IX73 inverted fluorescence microscope (Olympus, Tokyo, Japan). We used tissue sections from the small intestine of the infected lion as positive controls in each run, and sense-strand probes were applied to adjacent sections as negative controls. Prior to hybridization, adjacent sections were pretreated with RNase A to serve as an additional negative control.

### 2.6. Virus Isolation in Cell Culture

We attempted virus isolation from tracheal, kidney, small intestinal, and rectal tissues of the FeKoV0813-infected white lion using CRFK, F81, MDCK, and Vero E6 cells. Tissues were homogenized in Dulbecco’s modified Eagle’s medium (DMEM) with penicillin/streptomycin, clarified by centrifugation at 3000× *g* for 15 min, and filtered through 0.22 μm filters. The filtrates were used to inoculate cell monolayers maintained at 37 °C under 5% CO_2_. Cultures were maintained with or without 2 μg/mL trypsin, passaged blindly three times, and monitored for cytopathic effects (CPE) for 7 days after each passage. Supernatants were harvested at each passage and tested by qPCR [24,25].

### 2.7. Quantitative Analysis of Viral Expression with qPCR in Different Tissues

To quantify FeKoV RNA across tissues, an absolute qPCR assay was established using a plasmid-based standard curve targeting the *VP1* gene. A 208 bp *VP1* fragment was cloned into the pMD19-T vector (Takara Bio Inc., Kusatsu, Japan) to generate the standard plasmid. Plasmid concentration was determined spectrophotometrically, and the corresponding copy number was calculated from the plasmid molecular weight. Tenfold serial dilutions ranging from 10^1^ to 10^8^ copies per reaction were used to generate the standard curve. Total RNA extracted from each tissue was reverse-transcribed into cDNA using the PrimeScript RT Reagent Kit (Takara Bio Inc., Kusatsu, Japan) with random hexamer primers. Quantitative PCR was performed using SYBR Green Master Mix (Vazyme Biotech Co., Ltd., Nanjing, China) and specific primers (Supplementary Table S1) on a CFX Connect Real-Time PCR Detection System (Bio-Rad, Hercules, CA, USA). The cycling conditions consisted of an initial denaturation at 95 °C for 30 s, followed by 40 cycles of 95 °C for 5 s, 60 °C for 30 s and a final extension at 72 °C for 30 s. All reactions were performed in three independent technical replicates.

Viral RNA copy numbers were calculated by interpolation from the standard curve using the corresponding threshold cycle (Ct) values. To facilitate comparisons among tissues, viral loads were normalized to 100 ng of total RNA input and expressed as log_10_ copies per 100 ng of total RNA. Normalization to 100 ng of total RNA was chosen because this amount falls within the linear range of the assay and allows consistent comparison across tissues with different RNA yields.

### 2.8. Viral Genome Assembly and Phylogenetic Analysis

Raw sequencing reads were processed using Trimmomatic v0.39 to remove adapter sequences and low-quality bases. The resulting clean reads were assembled de novo using MEGAHIT v1.2.9 with default parameters. Putative viral contigs were identified by BLASTn and BLASTx searches against the NCBI non-redundant nucleotide and protein databases. The complete FeKoV genome was reconstructed by iterative mapping of sequencing reads to the candidate viral contig using Bowtie2 v2.4.5, followed by manual inspection and curation in Geneious Prime v2023.2. Open reading frames were predicted using ORF finder.

For phylogenetic analysis, representative reference sequences from the six recognized species within the genus *Kobuvirus* (Aichivirus A-F) were retrieved from GenBank. Three amino acid datasets were constructed for phylogenetic tree reconstruction: the complete polyprotein, the P1 protein, and the VP1 protein. Each phylogeny comprised 24 sequences, with alignment lengths of 2736 aa, 854 aa, and 254 aa, respectively (Supplementary Table S3). Sequencing reads covered 100% of the FeKoV0813 genome. Multiple sequence alignments were generated with MAFFT v7.520 using the L-INS-i algorithm. Maximum-likelihood trees were inferred in IQ-TREE v2.2.0 under the GTR + F + I + G4 substitution model selected by ModelFinder. Branch support was assessed with 1000 ultrafast bootstrap replicates. Trees were visualized and annotated using FigTree v1.4.4 and Adobe Illustrator.

### 2.9. Codon-Based Selection Pressure Analysis

The *VP1* coding sequences of FeKoV0813 and representative AiV-A strains were aligned using MAFFT v7.520 with the L-INS-i algorithm. The *VP1* alignment included sequences from the following host origins: feline (*n* = 17), human (*n* = 52), and rodent (*n* = 56). The alignment length was 762 bp, with gaps and ambiguously aligned positions removed. All sequences used are listed in Supplementary Table S4 with their accession numbers. The alignment was manually inspected to preserve codon integrity, and sequences containing internal stop codons or excessive gaps were excluded from subsequent analyses. Selection pressure was evaluated by estimating the ratio of nonsynonymous to synonymous substitution rates (dN/dS) using the Datamonkey server and four complementary methods implemented in the HyPhy framework: Single-Likelihood Ancestor Counting (SLAC) [26], Fixed-Effects Likelihood (FEL) [26], Mixed-Effects Model of Evolution (MEME) [27], and Fast Unconstrained Bayesian Approximation (FUBAR) [28]. Sites were considered significant at *p* < 0.1 for SLAC, FEL, and MEME, or a posterior probability ≥ 0.9 for FUBAR. To increase confidence in site-specific inferences, only residues supported by at least two methods were considered to show evidence of selection. To examine amino acid variation among host-associated AiV-A lineages, the VP1 amino acid sequence of FeKoV0813 was compared with representative human, feline, and rodent-associated strains to identify host-associated amino acid differences.

### 2.10. Recombination Analysis

To screen for potential recombination events in the lion-derived FeKoV0813 genome, we first performed an initial scan using the Recombination Detection Program (RDP) v4.95. The complete genome sequence of the lion FeKoV strain was used as the query against a reference panel of representative kobuvirus sequences retrieved from GenBank. Seven recombination detection algorithms implemented in RDP4 were applied: RDP, GENECONV, BootScan, MaxChi, Chimaera, SiScan, and 3Seq [29,30]. A putative recombination event was considered significant only if it was supported by at least six of these methods, with *p* < 0.05 after Bonferroni correction. Candidate breakpoints were identified based on the consensus positions from multiple algorithms.

### 2.11. Transcriptomic Analysis of Viral and Host Gene Expression

Clean reads from each tissue sample were aligned to the assembled FeKoV0813 reference genome using Bowtie2 v2.4.5 with default settings. Viral read abundance was normalized to the total number of mapped reads and reported as reads per million (RPM), enabling quantitative comparison of viral RNA abundance across tissues.

For host transcriptome analysis, clean reads were aligned to the lion reference genome (*Panthera leo*, GCF_018350215) using STAR v2.7.10a with the quantMode GeneCounts option. Gene expression levels were quantified as transcripts per million (TPM) using RSEM v1.3.3. The expression profiles of selected immune-related genes, including the pro-inflammatory cytokines *IL1A*, *IL1B*, *IL6* and *IL18* and the chemokines *CXCL8* and CXCL10, were extracted from the TPM matrix and compared across tissues.

### 2.12. Statistical Analysis

Given that this study was based on a single animal and the repeated samples represented technical rather than biological replicates, the data are presented descriptively as the mean ± standard deviation (SD) of the technical replicates; no inferential statistical tests were performed to compare different tissues. All statistical analyses were performed using R version 4.2.0.

## 3. Results

### 3.1. Clinical Presentation and Gross Pathology

Clinical records for this animal were incomplete because the keeper did not document the daily progression of illness, and no detailed timeline or further clinical progression is available; the available records indicate that the affected white lion exhibited coughing, anorexia, and watery diarrhea in the pre-death period and developed acute respiratory distress characterized by tachypnea, open-mouth breathing, and moist rales on thoracic auscultation. Antibiotic therapy and intravenous nutritional/fluid support were initiated immediately after these signs were observed, but despite supportive treatment, the animal’s condition deteriorated rapidly and culminated in death. At necropsy, the tracheal mucosa was markedly congested and covered by diffuse hemorrhagic exudate; segmental congestion and mild thickening of the intestinal wall were observed in the ileum, and the kidneys exhibited multifocal petechial hemorrhages, whereas no gross abnormalities were detected in the heart, lungs, liver, or pancreas.

### 3.2. Detection of Aichivirus a in the White Lion and Screening for Co-Infecting Pathogens

Metatranscriptomic sequencing, followed by qPCR validation, detected AiV-A nucleic acids in tissue samples collected from the white lion. To investigate the potential contribution of other infectious agents to the disease, the metatranscriptomic dataset was retrospectively screened for common feline pathogens, including feline herpesvirus 1 (FHV-1), feline parvovirus (FPV), feline coronavirus (FCoV), feline immunodeficiency virus (FIV), feline bocavirus (FBoV), feline leukemia virus (FeLV), feline rotavirus (FRV), *Mycoplasma felis*, *Bordetella bronchiseptica*, *Chlamydia felis*, and *Salmonella* [31]. No reads mapping to these pathogens were detected above the predefined background thresholds, providing no metatranscriptomic evidence for co-infection with these agents in this case. But *Salmonella* identification relied solely on sequencing without bacterial culture, representing a limitation.

### 3.3. Histopathology and In Situ Localization of FeKoV0813 in Tissues

**Figure 1:**
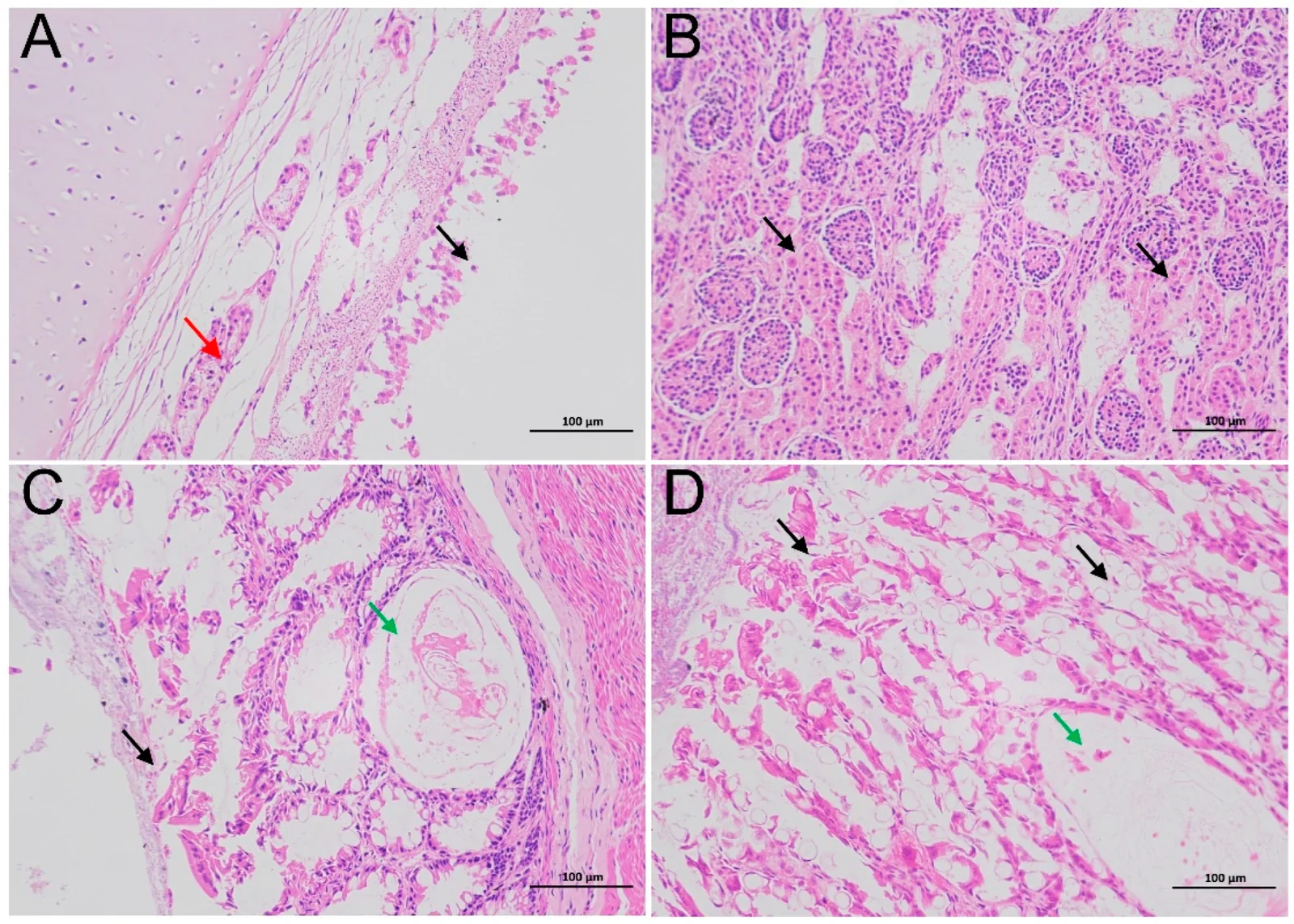
Histopathological findings in FeKoV0813-infected tissues. Representative H&E-stained sections of trachea, kidney, small intestine and rectum from the FeKoV0813-infected white lion. (**A**) In the trachea, extensive epithelial necrosis and shedding (black arrow) expose the underlying lamina propria, with necrotic glandular epithelial cells visible in the submucosa (red arrow). (**B**) In the kidney, cortical architecture is disorganized, with widespread necrosis and sloughing of tubular epithelial cells (black arrows); glomeruli are relatively unaffected. (**C**,**D**) In the small intestine and rectum, the mucosa shows extensive epithelial necrosis (black arrows), focal glandular dilatation (green arrows), epithelial flattening and intraluminal eosinophilic debris. Scale bars, 100 μm.

**Figure 2:**
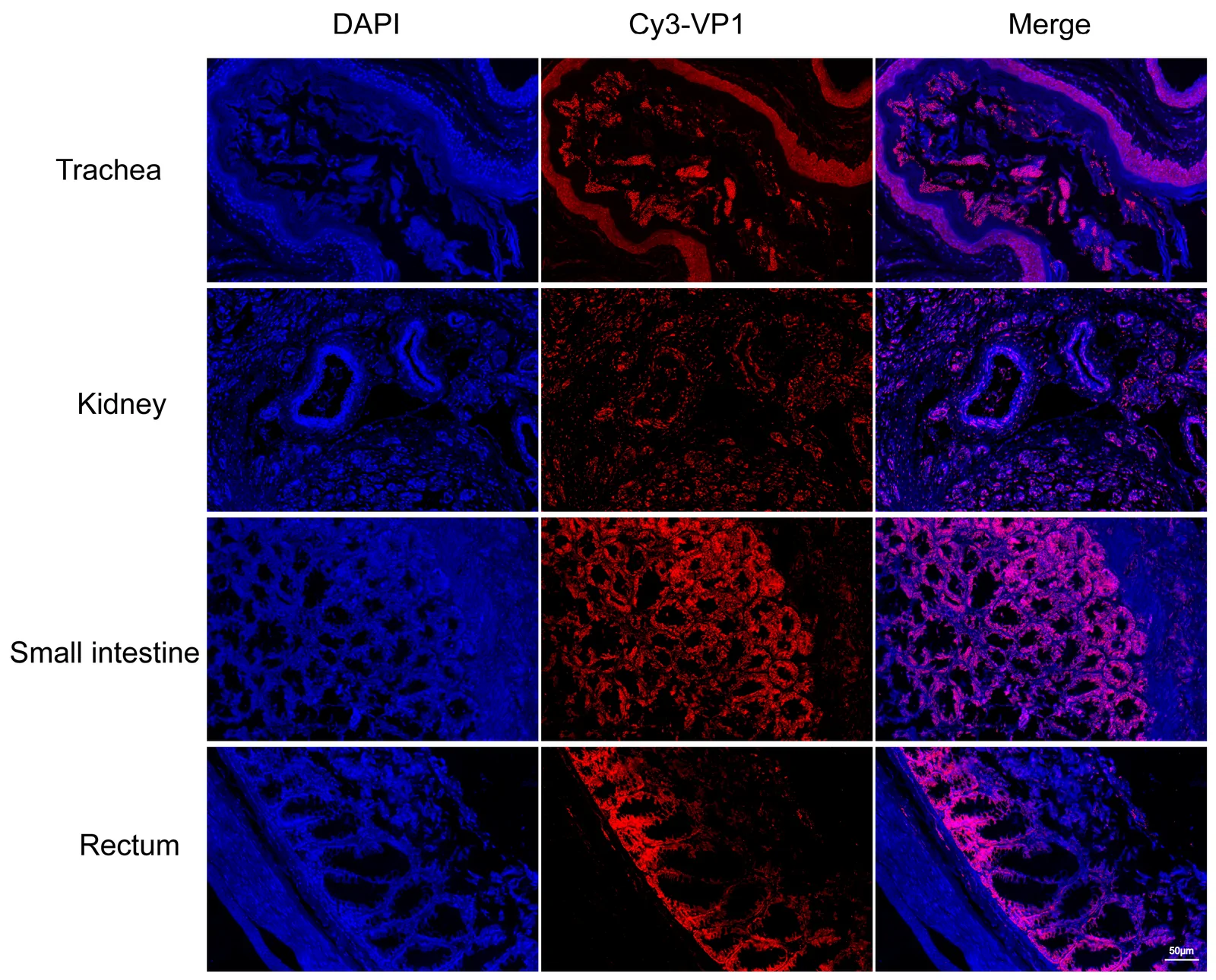
In situ localization of FeKoV0813 RNA in infected tissues. Dual-fluorescence in situ hybridization (FISH) was performed on sections of trachea, kidney, small intestine and rectum using a Cy3-labelled probe targeting the FeKoV *VP1* gene (red); nuclei were counterstained with DAPI (blue). Merged images show the spatial relationship between viral RNA and cellular nuclei. In the trachea, Cy3 signals are concentrated in the surface epithelium and submucosal glands. In the kidney, labelling is restricted to renal tubular epithelial cells in the cortical region, with no signal detected in glomeruli. In the small intestine, intense signals localize predominantly to villus tips. In the rectum, signals are confined to the surface epithelium. Scale bars, 50 μm.

We examined tissue pathology by H&E staining and localized FeKoV0813 RNA by dual-fluorescence in situ hybridization (FISH) targeting the viral *VP1* gene in the trachea, kidney, small intestine and rectum. In the trachea, H&E staining showed extensive epithelial necrosis and shedding, which exposed the underlying lamina propria; necrotic glandular epithelial cells were visible in the submucosa (Figure 1A). FISH detected prominent Cy3-labelled signals localized to the surface epithelium and submucosal glands, consistent with the high viral load quantified in this tissue (Figure 2). In the kidney, the cortical architecture was disorganized, with widespread necrosis and sloughing of tubular epithelial cells, whereas glomeruli were relatively unaffected (Figure 1B). Despite the absence of overt tissue damage, FISH detected distinct Cy3-positive signals in the cortical region, associated with renal tubular epithelial cells; glomeruli were consistently negative. In the small intestine and rectum, the mucosa showed extensive epithelial necrosis, focal glandular dilatation, epithelial flattening, and intraluminal eosinophilic debris (Figure 1C,D). In the rectum, similar epithelial changes were noted. FISH analysis showed Cy3-labelled signals concentrated in the mucosal epithelium, predominantly at villus tips in the small intestine and along the luminal surface in the rectum. Signal intensity was higher in the small intestine than in the rectum, correlating with the viral load gradient measured by qPCR. FISH data indicate only that viral RNA is localized to specific cell types, rather than confirming the presence of productive viral replication or direct cytopathic effects.

### 3.4. Viral Load in Different Tissues

**Figure 3:**
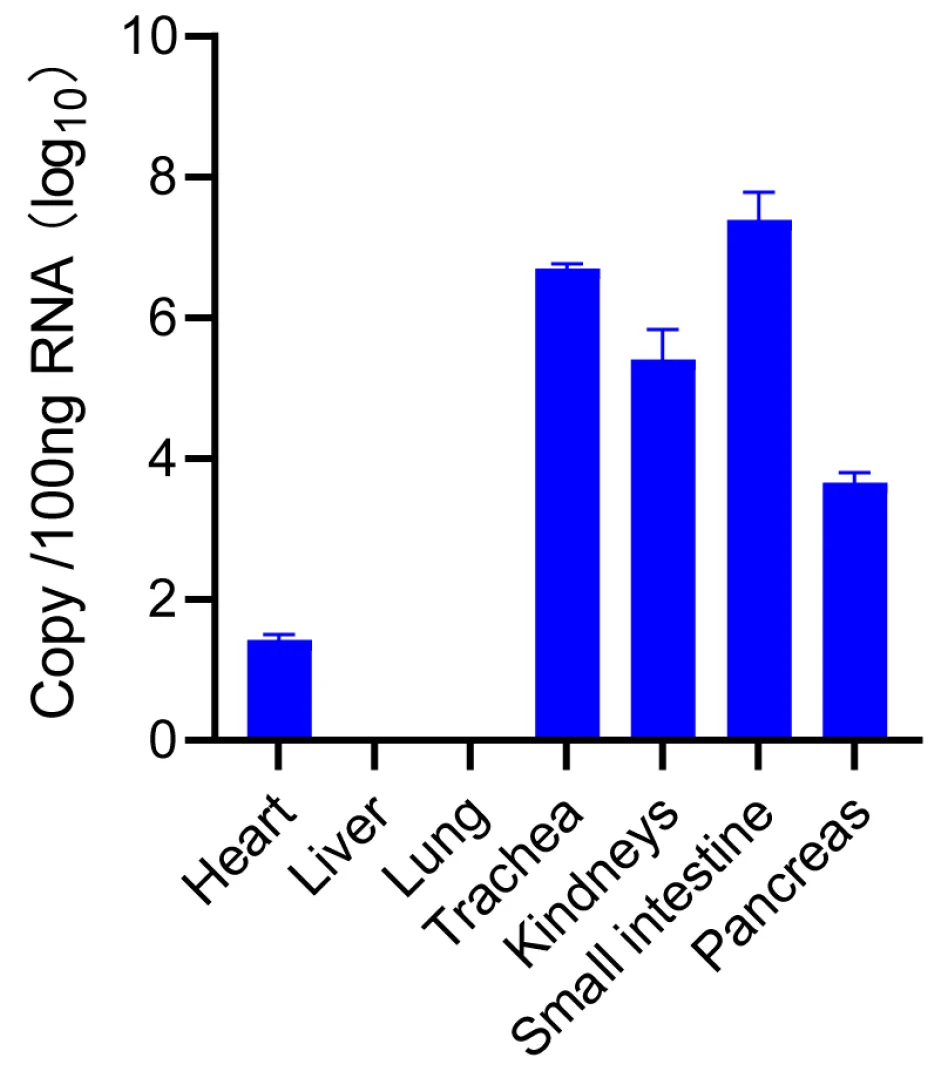
Tissue distribution of FeKoV0813 RNA. Viral RNA loads in seven tissues were quantified by absolute RT–qPCR. Viral RNA abundance was normalized to 100 ng of total RNA per reaction and expressed as log_10_ copies per 100 ng total RNA. Data are presented as mean ± s.d. from three independent technical replicates.

Absolute RT-qPCR quantified FeKoV0813 RNA in seven tissues (heart, liver, lung, trachea, kidney, small intestine, and pancreas; Figure 3). Assay performance was established with a 208 bp *VP1* plasmid standard (10^1^–10^8^ copies/reaction): efficiency was 98.7% (y = −3.35x + 39.82, R^2^ = 0.999), and the limit of detection (LOD) was 10 copies/reaction, respectively (Supplementary Figure S1A–C). Specificity was supported by a single melt peak at 82.5 °C (Supplementary Figure S1D–F), sequence confirmation of amplicons, and consistently negative no-template and uninfected feline tissue controls; representative Ct values ranged from 18.5 ± 0.3 (small intestine) to 33.2 ± 0.8 (heart), with negative tissues showing Ct > 40. Viral loads were normalized to 100 ng total RNA to allow comparison across tissues with differing RNA yields, as this amount falls within the assay’s linear range. FeKoV0813 RNA was detected in five of seven tissues: the highest load was in the small intestine (7.4 ± 0.5 log_10_ copies per 100 ng total RNA), followed by the trachea (6.7 ± 0.2), kidney (5.4 ± 0.4), pancreas (3.7 ± 0.2), and heart (1.3 ± 0.1); liver and lung remained negative.

The predominance of FeKoV0813 RNA in the small intestine and trachea, together with its detection in the kidney, pancreas, and heart, indicates a tissue distribution extending beyond the gastrointestinal tract. The absence of detectable viral RNA in the lung, despite severe clinical respiratory distress, is notable. This pattern may indicate that the respiratory signs were primarily attributable to tracheal pathology rather than direct pulmonary involvement. This interpretation is consistent with the high viral load and FISH signals in the trachea. The respiratory distress may also have been exacerbated by non-infectious factors or may have been secondary to systemic illness. Another possibility is that viral RNA in the lung was below the assay’s detection limit and could represent either genuine low-level dissemination or trace contamination from highly viremic blood present in the tissue.

### 3.5. Genomic Characterization and Phylogenetic Analysis of FeKoV0813

#### 3.5.1. Genomic Structure Characteristics

**Figure 4:**
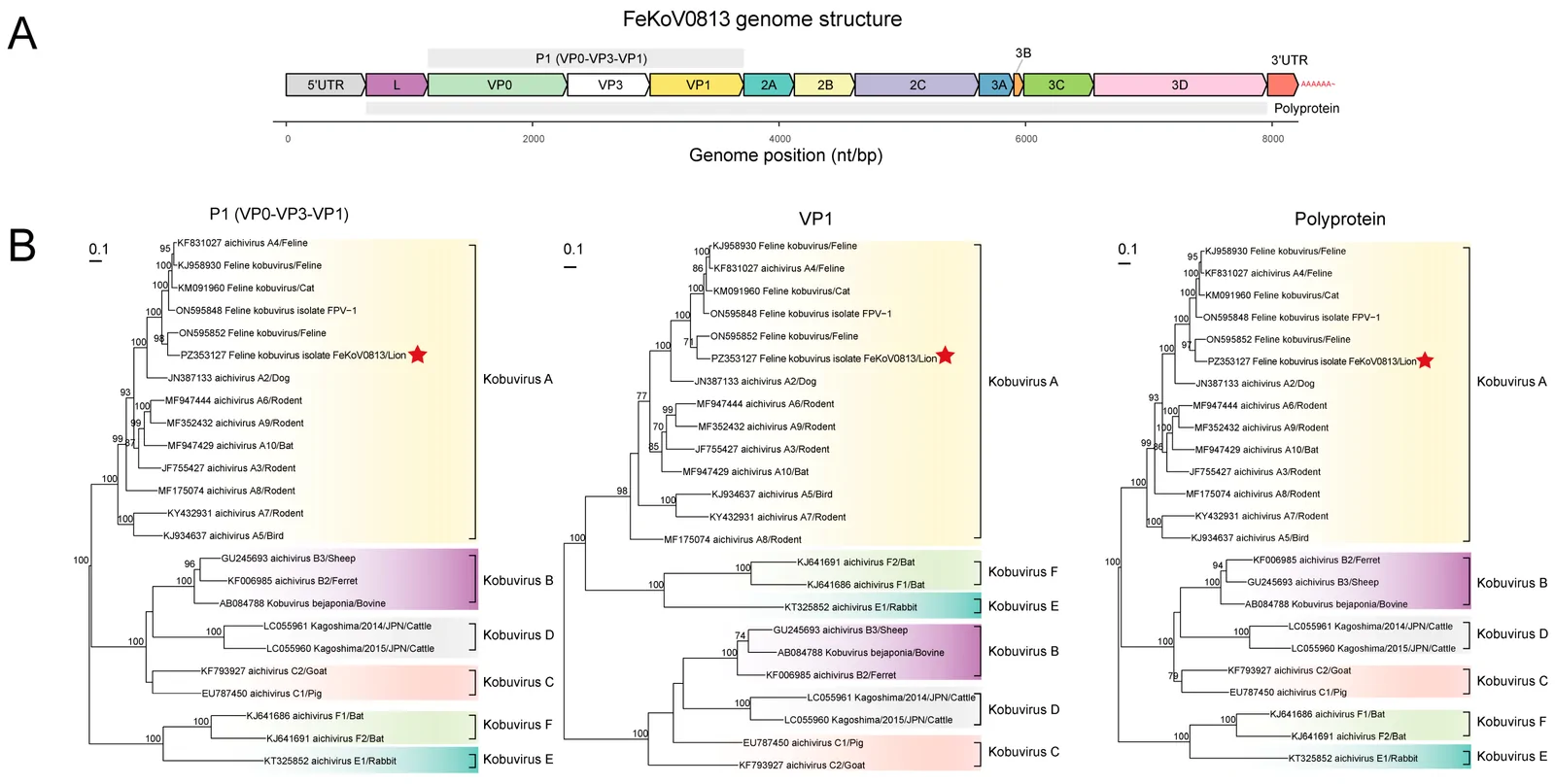
Genomic architecture and phylogenetic characterization of the lion-derived kobuvirus FeKoV0813. (**A**) Schematic of the FeKoV0813 genome, comprising a 5′ UTR, a single polyprotein-encoding ORF, and a 3′ UTR. The polyprotein is processed into the leader protein (L), structural capsid proteins (VP0, VP3, and VP1), and non-structural proteins (2A–2C, 3A–3D). (**B**) Maximum-likelihood phylogenies inferred with IQ-TREE (1000 ultrafast bootstrap replicates) based on the complete polyprotein, P1 protein, and the VP1 protein. The red star represents the strain used in this study.

The complete genome of FeKoV0813 was determined to be 8213 nucleotides (nt) in length, excluding the 3′ terminal poly(A) tail (Figure 4A). The genome exhibited a canonical Kobuvirus genomic organization, comprising a 5′ untranslated region (5′ UTR, 648 nt), a leader protein (L, 504 nt), a structural protein region (P1: VP0, VP3, and VP1, at 1131, 669, and 762 nt, respectively), two non-structural protein regions (P2: 2A, 2B, and 2C, at 408, 495, and 1005 nt, respectively; P3: 3A, 3B, 3C, and 3D, at 282, 81, 570, and 1404 nt, respectively), and a 3′UTR (251 nt). The GC content across distinct genomic regions varied from 50.6% to 61.7%, with the highest observed in the VP3 region (61.7%) and the lowest in the 3′UTR (50.6%), consistent with observations in other reported kobuviruses (Table 1) [32].

#### 3.5.2. Sequence Homology Analysis

To elucidate the genetic relatedness of FeKoV0813, pairwise nucleotide (nt) and deduced amino acid (aa) sequence identities were calculated against representative reference strains of Kobuvirus species A to F (Table 2). The result showed that FeKoV0813 shared the highest genetic identity with members of AiV-A. Within the AiV-A species, the closest relationship was observed with a feline-derived strain (Cat/FK13), with complete genome nt identity of 87.5%, and ORF nt/aa identities of 87.8% and 93.8%, respectively. Lower identities were observed with the rat-derived strain (Rat/Rt386), sharing 74.5% nt identity across the complete genome. At the structural protein level, VP0 and VP3 displayed notably high conservation (nt/aa identities of 84.0–93.4% and 87.993.8%, respectively), whereas VP1 was comparatively less conserved (81.8% nt and 71.6% aa). In contrast, identities with AiV-B to AiV-F strains were significantly lower, with complete genome nt identities ranging from 48.0% to 54.3% and ORF aa identities from 43.8% to 57.6%. These data strongly corroborate the classification of FeKoV0813 within *Kobuvirus* species A.

#### 3.5.3. Phylogenetic Analysis

Phylogenetic trees were reconstructed using complete polyprotein, P1 and VP1 amino acid sequences (Figure 4B). The complete polyprotein was used to infer the overall evolutionary position of FeKoV0813 within the genus *Kobuvirus*; P1 and VP1 were selected because they encode structural capsid proteins involved in host cell recognition and receptor binding and include the most surface-exposed, neutralizing antibody-targeted determinants of host range and antigenic diversity. Together, these datasets provide complementary insights into the evolutionary relationships and potential host adaptation of FeKoV0813. Across all three datasets, FeKoV0813 consistently clustered within the AiV-A clade, with strong bootstrap support (>99%). Within this clade, FeKoV0813 grouped with AiV-A strains detected in carnivorous mammals, including cats, dogs and foxes. The concordant phylogenetic placement across genomic regions, together with the sequence identity analyses, supports the classification of FeKoV0813 as a member of AiV-A. Its clustering with viruses from other carnivorous hosts is consistent with shared evolutionary relationships among these kobuviruses, although the available phylogenetic data alone do not establish cross-species transmission or host-specific co-evolution. No recombination events in lion kobuvirus or phylogenetically closely Aichi virus were detected.

#### 3.5.4. Selection Pressure Analysis

Selection pressure on the VP1 gene was assessed across feline-, human- and rodent-associated kobuvirus lineages using four codon-based methods implemented in HyPhy: SLAC, FEL, MEME and FUBAR. Sites were considered significant at *p* < 0.1 for SLAC, FEL and MEME, and at a posterior probability ≥ 0.9 for FUBAR.

No positively selected sites were identified in the feline lineage by any of the four methods (Supplementary Table S2). In contrast, evidence of pervasive purifying selection was detected, with FEL and SLAC identifying 79 and 6 negatively selected sites, respectively. These findings indicate that the analyzed feline kobuvirus *VP1* sequences are predominantly subject to purifying selection. Evidence of episodic positive selection was detected in the human and rodent lineages. MEME identified six candidate sites in human VP1, including site 216, which was also supported by FUBAR, whereas three candidate sites were detected in rodent VP1, with site 243 additionally supported by FEL and FUBAR. Because the analyses were restricted to internal branches, lineage-specific selection acting on terminal branches was not evaluated. The purifying selection signature of feline VP1 is derived from a relatively small dataset of feline AiV-A sequences (*n* = 17), and this limitation should be considered when interpreting the results. The conclusion may change as more feline kobuvirus genomes become available.

### 3.6. Viral Transcript Abundance and Host Immune Responses Across Tissues

**Figure 5:**
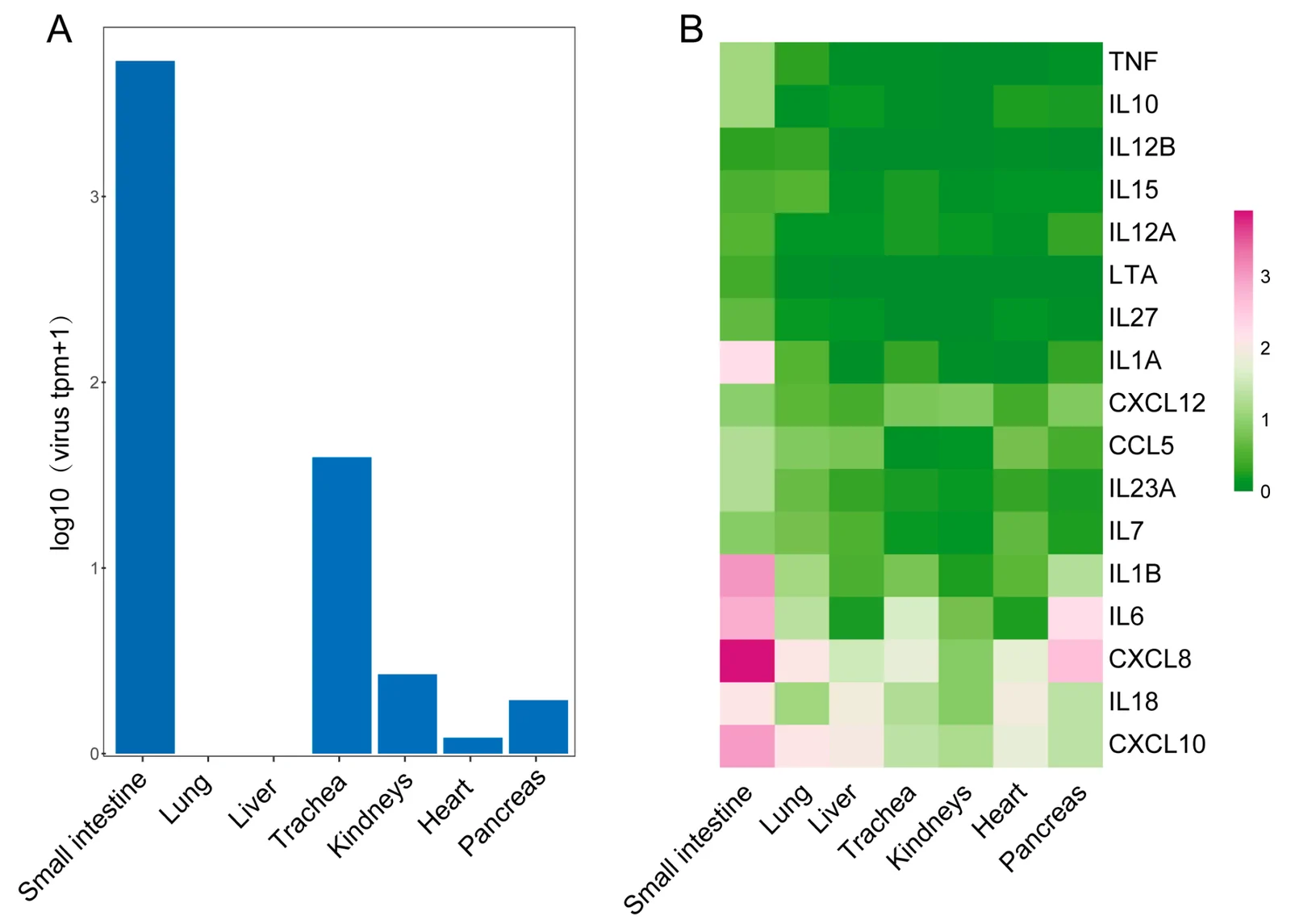
Viral transcript abundance and host immune gene expression profiles across tissues. (**A**) Viral transcript abundance in the small intestine, lung, liver, trachea, kidney, heart, and pancreas. Viral reads were normalized to the total number of mapped reads and are presented as log10(virus rpm + 1). (**B**) Heatmap of selected immune-related gene expression across the same tissues. The color scale indicates relative expression levels.

The relative abundance of kobuvirus-derived transcripts varied across the seven tissues examined (Figure 5A). Viral transcript abundance was highest in the intestine, followed by the trachea. Lower viral signals were detected in the kidney, pancreas and heart, whereas no viral transcripts were detected in the lung or liver. Among the immune-related genes examined, the intestine showed the highest transcript abundance of the pro-inflammatory cytokines *IL1A*, *IL1B*, *IL6* and *IL18* and the chemokines *CXCL8* and *CXCL10*. Of these genes, *CXCL8* exhibited the strongest transcriptional signal in the intestine (Figure 5B).

### 3.7. Viral Isolation Attempts

Infectious FeKoV was not recovered from tissue homogenates despite repeated isolation attempts using CRFK, F81, MDCK, and Vero E6 cells under different culture conditions, including incubation at 37 °C with or without trypsin. No cytopathic effects were observed after three subsequent blind passages, and RT-PCR of culture supernatants remained negative throughout.

## 4. Discussion

Kobuviruses have been identified in a broad range of mammalian hosts, including humans, cattle, swine, dogs, cats, sheep, rodents, and bats [1,2,5,10,33,34,35], but their biological and pathogenic significance in carnivores remains incompletely understood. In felids, current knowledge is derived almost exclusively from domestic cats, in which kobuviruses have been detected predominantly in fecal samples from animals with enteric disease [2,8,36]. The present case therefore extends the available evidence to a large felid and provides an opportunity to assess the tissue distribution and pathological relevance of AiV-A infection in a previously uncharacterized host. The intestinal distribution of FeKoV0813 is consistent with the predominantly enteric association reported for kobuviruses in domestic carnivores. Feline kobuviruses have been identified mainly in fecal samples from cats with diarrhea [7,12], although their contribution to disease has remained difficult to establish because most previous studies relied primarily on molecular detection rather than tissue-based analyses. In contrast, the present case provides convergent evidence from viral load quantification, in situ localization, and histopathology, supporting biologically relevant involvement of FeKoV0813 in the intestinal lesions.

The prominent tracheal involvement represents an important feature of this case. Kobuviruses in domestic carnivores are generally investigated in the context of enteric disease, and respiratory tract involvement has received comparatively little attention [7]. In the present case, histopathology, in situ hybridization, and qPCR-based viral load quantification together revealed high viral RNA loads in the trachea, in contrast to earlier reports that detected virus exclusively in the intestine. The preferential detection of FeKoV0813 RNA in the trachea, with no detectable viral RNA or gross lesions in the lung, may reflect cell type-specific constraints on viral entry or replication. Tracheal epithelium differs from alveolar epithelium in receptor repertoire, mucus composition, and innate immune signalling, and kobuviruses may exploit entry factors enriched in the upper airway. The tracheal mucosa may also support greater viral RNA accumulation than the distal lung. In this animal, respiratory distress was therefore more likely attributable to tracheal epithelial injury and inflammation, with associated luminal narrowing or obstruction, than to alveolar disease. Human rhinoviruses and foot-and-mouth disease virus (FMDV) both exhibit respiratory tropism, primarily infecting the upper respiratory tract, although they can also cause systemic disease in certain hosts [37,38]. The prominent tracheal involvement observed in our case, without detectable lung parenchyma invasion, may reflect a specific tropism for tracheal epithelial cells, consistent with the capacity of human rhinoviruses and FMDV to infect tracheal and bronchial epithelial cells in vivo and in primary culture [39]. Extra-intestinal detection and systemic distribution of kobuviruses have previously been reported in dogs and wild carnivores [4,40]. A previous study documented extraintestinal canine kobuvirus (CaKV) infection in a fox, with viral RNA detected in gastric epithelial cells, ventricular ependymal cells, lymph nodes, urinary bladder, trachea, and brain. This finding is consistent with our detection of viral RNA in the trachea, an extraintestinal site [17]. Together with the respiratory signs observed clinically, these findings support substantial involvement of the tracheal mucosa during infection. However, the presence of viral RNA alone does not demonstrate productive replication or direct viral injury. In this case, the concordance between viral load, tissue localization, and pathological changes provides the strongest evidence for viral involvement in the intestine and trachea.

FeKoV0813 clustered with carnivore-associated AiV-A strains and shared 87.8% and 75.6% whole-genome nucleotide sequence identity with the feline strain Cat/FK-13 and the rodent strain Rat/Rt386, respectively. In the antigenically critical VP1 region, it displayed 84.6%, 69.3%, and 32% amino acid sequence identity with the representative feline strain Cat/FK-13, the rodent strain Rat/Rt386, and the ferret strain Ferret/MpKoV38, respectively [6,36]. This relationship is consistent with the broad host range and genetic diversity reported for AiV-A [2,10]. Nevertheless, phylogenetic relatedness does not establish the source or direction of transmission. The present data therefore support a close evolutionary relationship between FeKoV0813 and other carnivore-associated AiV-A strains but do not determine whether the virus originated from domestic carnivores, wildlife, or another reservoir [16]. Therefore, in zoo settings, feral cats and dogs in and around zoological parks represent a continual risk of viral introduction, and effective prevention requires an integrated approach encompassing environmental hygiene, population management of stray animals, routine molecular surveillance, and biosecurity measures that restrict domestic carnivores from accessing felid enclosures. VP1 was more variable than VP0 and VP3, and selection-pressure analysis identified predominant purifying selection within the feline-associated VP1 lineage, in contrast to the episodic positive selection detected in the human and rodent lineages [41]. The predominance of purifying selection within the feline-associated VP1 lineage suggests that this capsid protein is subject to evolutionary constraint in these viruses. However, the limited number and host diversity of available sequences preclude conclusions regarding host adaptation or the evolutionary history of FeKoV0813 in large felids. As more feline kobuvirus genomes become available, this conclusion may need to be reassessed.

Transcriptomic profiling revealed marked upregulation of *CXCL8* and pro-inflammatory cytokines (*IL1A*, *IL1B*, *IL6*, *IL18*) in the intestine, with *CXCL8* exhibiting the strongest signal. This transcriptional pattern was consistent with the high intestinal viral burden and epithelial localization of FeKoV0813, suggesting a localized inflammatory response associated with infection. In feline viral infections, excessive neutrophil recruitment driven by *CXCL8* is strongly associated with mucosal barrier disruption, a mechanism well-documented in conditions such as feline infectious peritonitis and feline panleukopenia [42,43]. Alongside viral RNA detection in tracheal and renal epithelia, we hypothesize that this *CXCL8*-driven inflammatory burst compromised the mucosal integrity, thereby facilitating hematogenous seeding of FeKoV0813 to the kidney and pancreas [36,44]. However, this remains a hypothesis that requires further investigation. Transcriptomic analysis was based on tissues from a single animal and lacked uninfected white-lion controls. The observed transcriptional differences should therefore be interpreted as tissue-associated responses rather than as evidence of a defined pathogenic mechanism.

Host factors may also have contributed to disease progression. The potential immunological susceptibility of leucistic white lions, resulting from inbreeding-associated immune deficiencies, together with the stress of captivity and the juvenile age of the animal, may have increased susceptibility to severe disease. These factors could have acted synergistically with the high intestinal and tracheal viral RNA loads to produce the observed clinical severity. However, no immunological data were available, and this interpretation remains speculative.

The failure to isolate infectious FeKoV0813 is consistent with the known difficulty of culturing kobuviruses in vitro. Canine kobuvirus has likewise not been propagated in cell culture [45], and human Aichi virus is difficult to isolate, prompting the development of pseudovirus systems to study entry and replication [46]. A wild-type Korean AiV strain also failed to grow in Vero cells, attributed to predicted defects in viral RNA replication, including the absence of essential 5′ stem-loop structures [47,48]. In the present case, the cell lines used (CRFK, F81, MDCK, and Vero E6) may lack the receptor or cellular factors required for FeKoV0813 entry and replication; kobuviruses may recognize receptor-binding sites distinct from those of other picornaviruses [11], and cell susceptibility varies widely among lines. Viral titers may have fallen below the isolation threshold by the time of sampling, particularly if post-mortem collection occurred after peak shedding. Freeze–thaw processing and storage before inoculation may have further reduced infectivity, as such cycles can lower infectious titers of both enveloped and non-enveloped viruses [49]. Finally, the RNA detected by RT-qPCR and FISH may represent non-infectious remnants rather than replication-competent virions, since nucleic acid detection does not distinguish infectious from non-infectious particles. These possibilities do not negate the molecular, histopathological, and in situ evidence of FeKoV0813 RNA in multiple tissues, but they preclude confirmation of productive infection and, therefore, causality, revalence, host range, and pathogenic significance.

## 5. Conclusions

This study reports a single-case study describing the detection and genomic characterization of FeKoV0813, an AiV-A strain, in a captive juvenile white lion (*Panthera leo*) that died following severe gastrointestinal and respiratory disease. FeKoV0813 RNA was detected in multiple tissues, with the highest levels in the small intestine and trachea, and was localized to epithelial cells in both tissues. Phylogenetic analysis placed FeKoV0813 within a carnivore-associated AiV-A lineage, extending the known host range of AiV-A to a large felid. Given the single-case design and unsuccessful virus isolation, Koch’s postulates were not fulfilled and the contribution of FeKoV0813 to the observed disease and death cannot be determined. Further investigations are required to clarify the prevalence, tissue tropism, and pathogenic significance of AiV-A in captive and wild felids. Kobuvirus should therefore be included in the differential diagnosis of captive large felids with concurrent enteric and respiratory signs, and routine surveillance is warranted in zoological settings where contact with domestic carnivore reservoirs may occur.

## Figures and Tables

**Table 1:** The location and size information of each gene of the FeKoV0813 strain.

Gene		Location	Size/bp	GC%
5′UTR		1-648	648	56.9
*L*		649-1152	504	56.5
*P1*	*VP0*	1153-2283	1131	59.9
	*VP3*	2284-2952	669	61.7
	*VP1*	2953-3714	762	60.4
*P2*	*2A*	3715-4122	408	61.3
	*2B*	4123-4617	495	57.8
	*2C*	4618-5622	1005	57.7
*P3*	*3A*	5623-5904	282	59.6
	*3B*	5905-5985	81	58
	*3C*	5986-6555	570	59.8
	*3D*	6556-7959	1404	55.7
3′UTR		7963-8213	251	50.6

**Table 2:** Nucleotide (nt) and amino acid (aa) identities between FeKoV0813 and representative kobuvirus reference strains.

Shared nt Identity (%)/aa Identity (%)									
Gene Region	AiV-A Cat/FK-13	AiV-A Rat/Rt386	AiV-B Ferret/MpKoV38	AiV-B Sheep/TB3	AiV-C Pig/S-1	AiV-C Goat/12Q108	AiV-D Cattle/Kagoshima	AiV-E Bat/JX2010	AiV-F Bat/GD2012
	KF831027 nt/aa	MF352432 nt/aa	KF006985 nt/aa	GU245693 nt/aa	EU787450 nt/aa	KF793927 nt/aa	LC055961 nt/aa	KJ641686 nt/aa	KJ641691 nt/aa
5‘UTR	85.2/-	70.9/-	-/-	28.6/-	22.9/-	27.1/-	-/-	-/-	-/-
*L*	86.1/88.7	52.1/49.7	31.7/26.8	34/25	31.8/26.7	33.1/23.6	32.1/25.3	29.6/9.3	31.1/0
*VP0*	84/93.4	74.9/83.6	61.8/59.9	59.8/59.9	60.5/60.4	63.5/64.2	52.4/50.3	48.4/47.6	47.8/47
*VP3*	87.9/93.8	78.4/83.9	65.8/58	66.5/58.5	63.4/59.4	62.1/59.8	58.8/50.4	64.9/65.2	62.1/65.2
*VP1*	81/84.6	71.6/69.3	43.6/32	42/32.7	43.6/36.8	44.6/33.6	40.9/33.1	41.2/34.2	42/31.9
*2A*	91.6/96.4	75.4/70.3	57.7/55.4	55.9/58	55.9/53.6	54.7/53.6	56.2/54	46.2/36.4	48.6/33.6
*2B*	92.1/98.8	80.6/82.4	47.6/50.3	46.7/52.1	49.6/45.6	45.8/54.5	43.4/38.5	40.3/29.6	40.2/32.8
*2C*	90.9/97.6	80.9/89.9	67.6/69	65.8/68.2	64/67.9	63.6/67.9	58.4/55.1	53.4/48.7	50/47.2
*3A*	89.4/97.6	71.9/89.9	47.7/69	46.4/68.2	52.7/67.9	50.5/67.9	48.8/55.1	37.2/48.7	35.9/47.2
*3B*	90.1/96.3	76.5/77.8	38.7/50	40.9/53.3	33.3/35.3	37.3/38.2	25/31.3	29.2/16.7	14.4/25.9
*3C*	87.9/94.7	75.8/81.1	57.2/49.8	53.8/48.7	53.5/52.1	57.8/53.1	54.1/46.9	41.3/34.9	41.6/33.2
*3D*	90.2/95.5	81.2/89.3	70.9/74.8	70.7/74.8	70.4/74	68.3/73.8	66/68.5	62.2/62.2	60.2/60.5
3‘UTR	87.6/-	60.5/-	18.3/-	21.1/-	18.1/-	19.1/-	19.7/-	29/-	25.2/-
complete	87.5/-	74.5/-	54.3/-	53.5/-	53.1/-	53.4/-	50.7/-	48.2/-	48/-
ORF	87.8/93.8	75.6/80.3	57.6/55.5	56.7/55.4	56.6/55.4	56.7/56	52.5/49	48.9/42.1	48.7/43.8

“-” indicates that it does not apply to identity comparison.

## Data Availability

The complete genome sequence of the FeKoV strain reported in this study has been deposited in GenBank under accession number [PZ353127]. The transcriptomic data have been deposited in the NCBI Sequence Read Archive under BioProject accession number PRJNA1510436. https://www.ncbi.nlm.nih.gov/bioproject/PRJNA1510436 (accessed on 21 July 2026).

## Supplementary Materials

The following supporting information can be downloaded at: https://www.mdpi.com/article/10.3390/microorganisms14102256/s1, Figure S1: Amplification and melt curve analysis of the quantitative PCR (qPCR) assay. (A–C) Amplification curves obtained from 10-fold serial dilutions of the standard template ranging from 10^1^ to 10^8^ copies per reaction. The X-axis represents the cycle number, and the Y-axis represents the relative fluorescence units (RFU). (D–F) Melt peak analysis of the corresponding qPCR products. A single, sharp melting peak was observed at approximately 82.5 °C for all concentrations, indicating high primer specificity and the absence of primer-dimers or non-specific amplifications. Data are representative of three independent replicates.; Table S1: FeKoV0813 The primers used in this study; Table S2: Transcriptome Sequence Alignment Rate; Table S3: Sequences used for phylogenetic tree reconstruction of the complete polyprotein, P1, and VP1 proteins of kobuviruses; Table S4: VP1 coding sequences used for selection pressure analysis of AiV-A strains from feline, human, and rodent hosts.
