# Supplementary material for: Aichivirus a Infection Associated with Severe Enteric and Respiratory Disease in a Captive White Lion (Panthera leo)
Source: Microorganisms. 2026 Oct 4;14(10):2256. doi: 10.3390/microorganisms14102256 (PMC13707751; doi:10.3390/microorganisms14102256)
Supplement: Supplementary file 1 [file microorganisms-14-02256-s001.zip › microorganisms-4538427-supplementary/Supplementary Tables S1 and S2 and Figure S1.pdf]

Supplementary Table S1. FeKoV0813 The primers used in this study.

Primer name	Sequences (5′-3′)	Location	PCR Product size
qPCRkubo-F	ACTGCCATGCTCTCCTGCTTT	3220-3240	208bp
qPCRkubo-R	ATGAAATGAGAGTTGCATTAG	3407-3427	
Kubu-Probe	CCAGACCAGTCCTCCCACCCGTAG	3474-3497	

Supplementary Table S2. Transcriptome Sequence Alignment Rate.

Library	Assigned reads	Ambiguous reads	Kobuvirus (TPM)
Small intestine	35,715,962	1,468,708	25,664.92
Lung	39,348,817	635,599	0
Liver	42,011,153	1,409,268	0
Trachea	33,825,845	1,300,235	174.9487
Kindney	36,178,023	2,911,787	10.3412
Heart	56,692,328	2,221,284	1.5633
Pancreas	30,894,602	1,262,694	4.0112

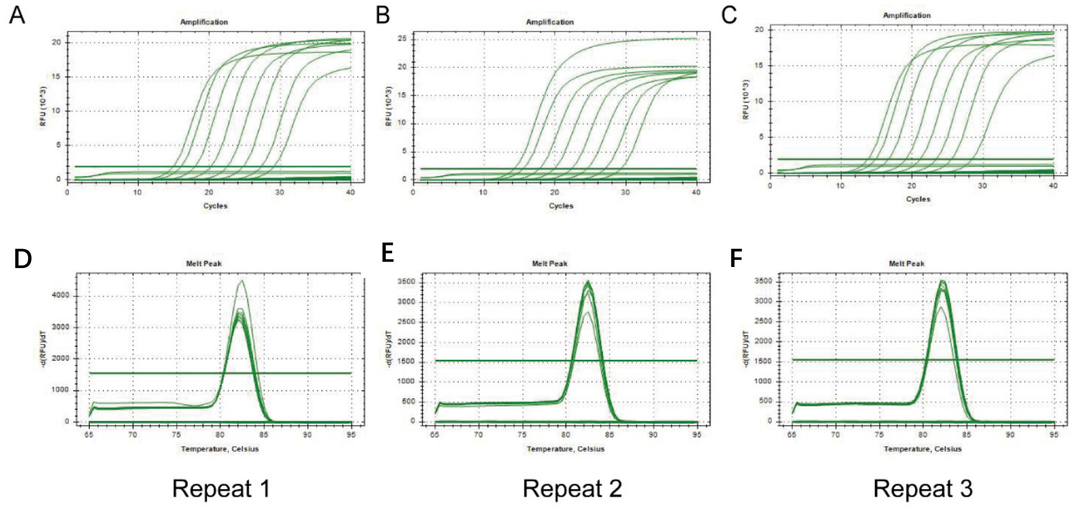

Supplementary Figure S1. Amplification and melt curve analysis of the quantitative PCR (qPCR) assay. (A–C) Amplification curves obtained from 10-fold serial dilutions of the standard template ranging from 10^1 to 10^8 copies per reaction. The X-axis represents the cycle number, and the Y-axis represents the relative fluorescence units (RFU). (D–F) Melt peak analysis of the corresponding qPCR products. A single, sharp melting peak was observed at approximately 82.5°C for all concentrations, indicating high primer specificity and the absence of primer-dimers or non-specific amplifications. Data are representative of three independent replicates.
